# Association of Protein Intake in Three Meals with Muscle Mass in Healthy Young Subjects: A Cross-Sectional Study

**DOI:** 10.3390/nu11030612

**Published:** 2019-03-13

**Authors:** Jun Yasuda, Mai Asako, Takuma Arimitsu, Satoshi Fujita

**Affiliations:** Faculty of Sport and Health Science, Ritsumeikan University, 1-1-1 Nojihigashi, Kusatsu, Shiga 525-8577, Japan; 55fhyanh@gmail.com (J.Y.); achaco0512@gmail.com (M.A.); arimitsu@fc.ritsumei.ac.jp (T.A.)

**Keywords:** young subjects, cross-sectional study, muscle mass, fat-free mass, appendicular fat-free mass, dietary records, protein intake, DXA, dual-energy X-ray absorptiometry

## Abstract

Protein intake of >0.24 g/kg of body weight (BW) at a single meal is necessary to maximize muscle protein synthesis in a young population. However, the association between the protein intake rate for three meals and muscle mass in the young population has not been evaluated. We hypothesized that a protein intake of >0.24 g/kg BW at all three meals is effective for maintaining muscle mass. Therefore, we cross-sectionally examined the association between protein intake at all three meals with muscle mass in 266 healthy young subjects (aged 21.4 ± 2.4 years). Subjects were divided into the AP group, which achieved protein intake >0.24 g/kg BW at all three meals; and the NP group, which did not. We calculated total fat-free mass (FFM) and appendicular fat-free mass (AppFFM) with dual-energy X-ray absorptiometry, and the percentage of total FFM (TotalFFM%) and appendicular FFM (AppFFM%) were calculated as the percentage of BW (%BW). We demonstrated that TotalFFM% (77.0 ± 0.5 vs. 75.2 ± 0.4%, *p* = 0.008) and AppFFM% (34.7 ± 0.3 vs. 34.1 ± 0.2%, *p* = 0.058) were higher in the AP than in the NP group. This finding suggests that achieving protein intake of >0.24 g/kg BW at all three meals is important for muscle mass maintenance in young populations.

## 1. Introduction

Maintaining and increasing muscle mass is important for health improvement, for example through the prevention of metabolic syndromes [1], diabetes [2], and sarcopenia [3]. Moreover, for athletes or people who like to exercise, regulation of muscle mass can be also an essential aspect for improvement of their performance [4,5,6].

Protein intake is well-known as one of the important elements for the regulation of muscle mass. Muscle mass is mainly regulated by net protein balance between muscle protein synthesis (MPS) and muscle protein breakdown (MPB); the augmentation of MPS over MPB results in muscle hypertrophy. A previous cross-sectional study has reported that total protein intake, calculated by the energy-adjusted intake by the residual method [7], was positively associated with fat-free mass (FFM) of the legs after adjusting for relevant confounders such as age, body mass index (BMI), and physical activity, in both adult men and women (aged 26–86 years) [8]. Moreover, a randomized controlled trial (RCT) conducted by Bray et al. set the following three groups: diets comprising 5%, 15%, and 25% of energy intake from protein sources [9]. In that study, each group that was involved in an overeating trial for 8 weeks without any exercise program, showed significantly increased body weight and fat mass. Interestingly, the groups with 15% and 25% energy intake also significantly gained total FFM, while the group with 5% energy intake experienced a 0.7 kg reduction in FFM [9]. These studies thus emphasize that total protein intake is important for the regulation of muscle mass regardless of habitual exercise, which is a well-established factor for the regulation of muscle mass. In addition, the standard daily protein intake in free-living conditions, known as the recommended dietary allowance (RDA), has been identified to have a positive relationship with FFM, thus demonstrating that daily protein intake greater than the RDA was required for maintaining muscle mass [10,11,12,13]. This suggests that the total daily intake of protein is important in the regulation of muscle mass in free-living conditions.

Recently, in addition to the importance of total protein intake, the protein intake from single protein consumption has been focused on in terms of the stimulation of MPS, which is an important factor for the regulation of muscle mass. Moore et al. [14] have reported that protein intake of 0.24 g/kg body weight (BW) was necessary to maximize MPS from single protein consumption in the young population. That study suggests that the fixed amount of high-quality protein intake (e.g., 0.24 g/kg BW) is required at the three traditional meals (breakfast, lunch, and dinner) to maximize MPS throughout the day. Consequently, achieving 0.24 g/kg BW of protein intake at three meals can be effective for maintaining muscle mass. On the contrary, inadequate protein intake over three meals may have negative consequences on muscle mass. According to the report that appendicular FFM (AppFFM) is a strong predictor of muscle mass [15], we therefore aimed to examine whether achieving 0.24 g/kg BW of protein intake at three meals (breakfast, lunch, and dinner) is associated with AppFFM in a healthy young population. In addition, to examine our purpose, because we have to consider the reported association of total protein intake with FFM [8,9], we also assessed whether there is an association between AppFFM and total protein intake using RDA values in our population.

## 2. Materials and Methods

### 2.1. Study Population

This cross-sectional study was conducted between July and September 2017. A total of 266 healthy college and graduate school students (aged 21.4 ± 2.4 years; 149 men, 117 women) who did not belong to a sports club participated in the present study. We collected 3-day dietary records to assess dietary intake. Using these records, the dietary intake of the subjects was assessed on two weekdays and one weekend day. After the recording, the subjects completed self-reported questionnaires and underwent anthropometric measurements. For any missing information or ambiguous answer, the subject was approached in person for clarification by a researcher. Our study was approved by the Ethics Committee for Human Experiments at Ritsumeikan University (BKC-IRB-2017-008) and was conducted in accordance with the Declaration of Helsinki. Informed consent was obtained from all subjects.

### 2.2. Anthropometric Measurements

Body mass index (BMI) was obtained by dividing the body weight by the square of the respective subject’s height (weight/height^2^ (kg/m^2^)). A radiological technician analyzed FFM and fat mass using dual-energy X-ray absorptiometer (DXA; Lunar Prodigy, GE Healthcare, Tokyo, Japan) with subjects in the supine position. From total body scans, we used enCORE version 15 software (GE Medical Systems Lunar, Madison, WI, USA), which generated automated measurements of FFM (arms, legs, and total body) and body fat percentage. Then, we calculated AppFFM from the data derived from measurements of the arms and legs. In addition, we calculated the percentage of total FFM (TotalFFM%) and AppFFM (AppFFM%) using the data of body weight as the main outcome to evenly compare these indices among individuals. This calculation of TotalFFM% and AppFFM% was according to the highest correlations between body weight and AppFFM in both men and women, as compared with height and BMI.

### 2.3. Dietary Assessment

The 3-day dietary records were photographed using digital cameras (DIGITAL CAMERA FinePix AX600, FUJIFILM, Tokyo, Japan) to improve the accuracy of dietary assessment and record the exact meal time. The dietary records included the following instructions: (1) “Please note your dietary records on 2 weekdays and 1 weekend day”, (2) “Please note all foods you had including confectionary or beverages”, (3) “Please take pictures of foods or nutrition facts, if it is cooked or processed food, before you eat”, and (4) “Please note your dietary records by referring to the examples provided.” In addition, the subjects were not restricted to record on either nonconsecutive or consecutive days to enable them to note their dietary records on the usual meal days. Photographic data of the 3-day records were collected and confirmed by a registered dietitian via face-to-face interviews with the subjects. The data were analyzed with Excel Eiyokun (version 8, Kenpakusha Co., Tokyo, Japan) based on the Standard Table of Foods Composition in Japan 2015.

### 2.4. Self-Reported Questionnaires

Self-reported questionnaires were used to collect information on lifestyle [living conditions (alone or with family), smoking and drinking habits], sleep quality according to the Pittsburgh Sleep Quality Index (PSQI), circadian rhythm type using the Morningness–Eveningness Questionnaire (MEQ), and physical activity using the International Physical Activity Questionnaire (IPAQ).

These variables could affect the results because they are associated with daily protein intake. Previous studies showed that people having smoking habit [16,17] or drinking habit [18,19] reduced daily protein intake. Individuals with poor sleep quality (higher PSQI score) had greater energy intake and a lower percentage of energy from protein [20]. In addition, individuals with evening activity preference (lower MEQ score) had a lower percentage of energy from protein, while energy intake was not significantly different from those with morning activity preference [21]. Furthermore, higher IPAQ score was associated with greater FFM [22].

#### 2.4.1. Pittsburgh Sleep Quality Index (PSQI)

The Japanese version of the PSQI indicates subjective sleep quality over the preceding month. The PSQI includes 7 components (range of subscale scores, 0–3): sleep quality, sleep latency, sleep duration, habitual sleep efficiency, sleep disturbance, use of sleeping medication, and daytime dysfunction [23]. A higher PSQI score implies lower subjective sleep quality. We also calculated bedtime, waking time, time in bed, and sleep quality (sleep duration/time in bed × 100) from the PSQI components.

#### 2.4.2. Morningness–Eveningness Questionnaire (MEQ)

The Japanese version of the MEQ evaluates self-rated preference for activity in the morning or evening [24,25]. The MEQ consists of 19 items on daily sleep habits or preferences (each score ranged 4–5 points). The total MEQ score (ranged 16–86 points) is then calculated. A lower MEQ score shows a preference to be active in the evening.

#### 2.4.3. International Physical Activity Questionnaire (IPAQ)

The Japanese version of the IPAQ comprises physical activity of 3 intensity levels (walking, moderate activity, and vigorous activity) and estimated time spent sitting per week, while the questions about time spent sitting were developed as separate indicators and not as part of the total physical activity scores [26,27]. The data were assessed for total weekly physical activity by weighting the reported minutes per week.

### 2.5. Statistical Analyses

**Figure 1:**
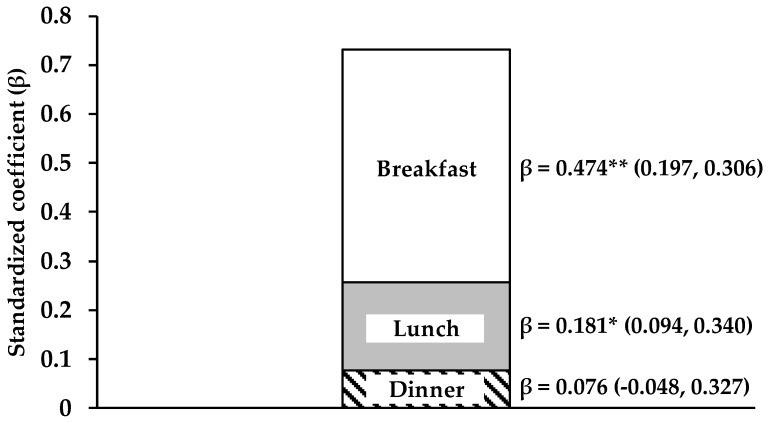
Contribution of achieving 0.24 g/kg BW of protein intake at each meal to total protein intake in all subjects. Multivariate regression analysis was used to examine the association of achieving 0.24 g/kg BW of protein intake at breakfast, lunch, and dinner (0: not achieved, 1: achieved, respectively) with total protein intake. Standardized coefficient (β) was obtained with adjustment for age, sex, drinking habit, smoking habit, living condition (alone or family), PSQI, MEQ, and IPAQ scores, and total energy intake with residual methods ^7^. ^*^: *p* < 0.05, ^**^: *p* < 0.001. Abbreviations: BW, Body Weight; PSQI, Pittsburgh Sleep Quality Index; MEQ, Morningness–Eveningness Questionnaire; IPAQ, International Physical Activity Questionnaire.

**Figure 2:**
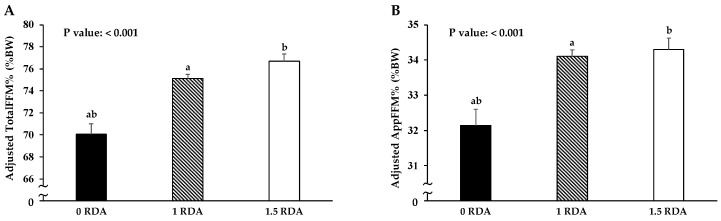
Comparisons of adjusted body compositions among the three groups (■: 0 RDA group, <0.9 g/kg BW, *n* = 30; ▧: 1 RDA group, 0.9–1.34 g/kg BW, *n* = 174; ☐: 1.5 RDA group, ≥ 1.35 g/kg BW/day of total protein intake, *n* = 62) in (**A**) adjusted TotalFFM% and (**B**) adjusted AppFFM%. Values are expressed as means ± SE, and adjusted for age, sex, drinking habit, smoking habit, living condition (alone or with family), PSQI, MEQ, and IPAQ scores, and total energy intake with residual methods [7] (post hoc analysis with Bonferroni correction; the same letters (a and b) indicating significant difference, *p* < 0.001). Abbreviations: RDA, recommended dietary allowance; BW, Body Weight; TotalFFM, total fat-free mass; AppFFM, appendicular fat-free mass; BW, body weight; PSQI, Pittsburgh Sleep Quality Index; MEQ, Morningness–Eveningness Questionnaire; IPAQ, International Physical Activity Questionnaire.

**Figure 3:**
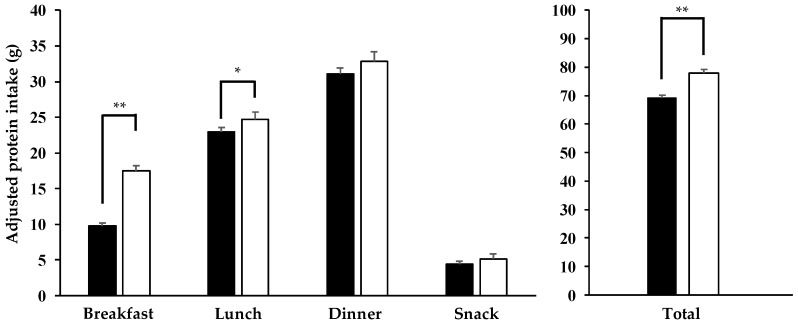
Adjusted protein intake at each meal and in total between the AP (☐: *n* = 83) and NP (■: *n* = 153) groups. AP group, achieving over 0.24 g/kg BW of protein intake at all three meals; NP group, not achieving 0.24 g/kg BW of protein intake from at least one meal. Values are expressed as means ± SE, and adjusted for age, sex, drinking habit, smoking habit, living condition (alone or with family), PSQI, MEQ, and IPAQ scores, and total energy intake with residual methods [7]. *: *p* < 0.05, **: *p* < 0.001. Abbreviations: BW, Body Weight; PSQI, Pittsburgh Sleep Quality Index; MEQ, Morningness–Eveningness Questionnaire; IPAQ, International Physical Activity Questionnaire.

**Figure 4:**
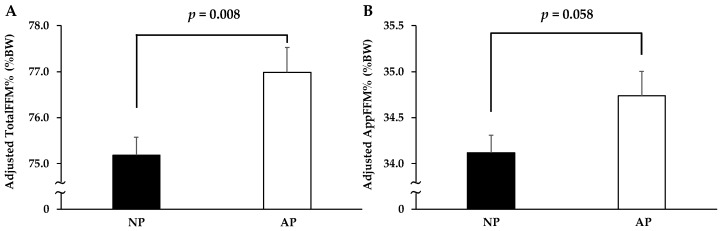
Comparisons of adjusted body compositions between AP (☐: *n* = 83) and NP (■: *n* = 153) groups with total protein intake more than RDA. After adjusting energy intake with residual methods ^7^, the subjects were separated into the two groups: the AP group, achieving over 0.24 g/kg BW of protein intake at all three meals; and the NP group, not achieving 0.24 g/kg BW of protein intake in at least one meal. Values are expressed as means ± SE, and adjusted for age, sex, drinking habit, smoking habit, living condition (alone or family), PSQI, MEQ, and IPAQ scores. Abbreviations: RDA, recommended dietary allowance; BW, Body Weight; PSQI, Pittsburgh Sleep Quality Index; MEQ, Morningness–Eveningness Questionnaire; IPAQ, International Physical Activity Questionnaire.

In order to remove the effect of total energy intake on total protein intake (Figure 1, Figure 2 and Figure 3) total protein intake was calculated with the use of the nutrient residual energy-adjusted method by regressing protein intake of individuals on their total energy intake [7]. In addition, we used the same residual method for protein intake at each meal with energy intake at the same meal (Figure 1, Figure 3 and Figure 4).

According to the previous study demonstrating the importance of 0.24 g/kg BW of protein intake for maximization of MPS [14] and the results of dietary intakes in the present study, we divided the subjects into two groups: the AP group, with subjects who achieved over 0.24 g/kg BW of protein intake at all three meals (breakfast, lunch, and dinner); and the NP group, with the subjects who did not achieve 0.24 g/kg BW of protein intake in at least one meal out of three main meals. Moreover, total protein intake was categorized based on the RDA for adults in Japan (0.9 g/kg BW/day) into groups of 0 RDA, 1 RDA, and 1.5 RDA (<0.9, 0.9–1.35, and ≥1.35 g/kg BW/day, respectively).

All values are displayed as mean± standard deviation (SD) or standard error (SE) (only for the results of analysis of covariance (ANCOVA)) for continuous variables or number (%) for categorical variables. The Mann–Whitney *U* test and the chi-squared test were applied to compare variables between sexes (Table 1 and Table 2). Multivariate regression analysis was used to examine the association of achieving 0.24 g/kg BW of protein intake at breakfast, lunch, and dinner (0: not achieved, 1: achieved, respectively) with total protein intake (Figure 1). ANCOVA was used to compare variables between groups with adjustment for confounders (Figure 2, Figure 3 and Figure 4). In addition, the confounders used in Figure 1, Figure 2, Figure 3 and Figure 4 are age, sex, drinking habit, smoking habit, living condition (alone or family), PSQI, MEQ, IPAQ scores, and total energy intake.

All statistical analyses were performed using SPSS version 23.0 for Windows (IBM Corp., Tokyo, Japan). *p* values < 0.05 using two-tailed tests were considered statistically significant.

## 3. Results

### 3.1. Subject Characteristics

Subject characteristics are shown in Table 1. Men had a significantly higher prevalence of drinking habit, weight, BMI, TotalFFM, AppFFM, TotalFFM%, AppFFM%, IPAQ score, and a later waking and dinner time compared with women, while women had significantly higher body fat percentage. In men, total energy, macronutrient intakes, and protein intake/kg body weight were significantly higher than in women (Table 2). Additionally, in men, energy and macronutrient intakes at lunch and dinner were significantly higher than in women, while energy and macronutrient intakes at breakfast and snack did not differ between sexes.

Furthermore, subject characteristics between AP and NP groups are shown in Tables S1 and S2 for men and women, respectively. Also, total dietary intake and dietary intake at each meal between AP and NP groups are illustrated in Tables S3 and S4 for men and women, respectively.

### 3.2. Protein Intake per Meal for Maximization of MPS

Based on a previous study indicating the necessity of protein intake over 0.24 g/kg BW for maximization of MPS [14], men had significantly greater protein intake per meal compared with women (15.8 ± 2.1 vs. 12.5 ± 1.4 g, *p* < 0.001). At dinner, the number of men achieving 0.24 g/kg BW of protein intake was significantly higher than that of women (96.6% (144/149 men) vs. 87.2% (102/117 women), *p* = 0.005). There were no significant associations of the number of subjects achieving 0.24 g/kg BW of protein intake with the sexes at: breakfast (33.6 (50/149 men) vs. 34.2% (40/117 women), *p* = 0.585) and lunch (87.9 (131/149 men) vs. 85.5% (100/117 women), *p* = 1.000).

### 3.3. Contribution of Achieving 0.24 g/kg BW of Protein Intake at Each Meal to Total Protein Intake

After adjusting for energy intake with residual methods [7], we performed multivariate regression analysis to examine the association of achieving 0.24 g/kg BW of protein intake at breakfast, lunch, and dinner with total protein intake. Figure 1 indicates that the contribution of achieving 0.24 g/kg BW of protein intake at breakfast to total protein intake was the highest (β = 0.474; Confidence Interval (CI): 0.197, 0.306; *p* < 0.001), that at lunch (β = 0.181; CI: 0.094, 0.340; *p* = 0.001) was the second highest, and that at dinner (β = 0.076; CI: −0.048, 0.327; *p* = 0.144) was the lowest, after adjusting for age, sex, drinking habit, smoking habit, living condition (alone or with family), and PSQI, MEQ, and IPAQ scores.

### 3.4. Association between FFM and RDA for Protein Intake

After adjusting for total energy intake with residual methods [7], Figure 2 displays the association of TotalFFM% and AppFFM% with groups of 0 RDA (<0.9 g/kg BW/day of total protein intake, *n* = 30), 1 RDA (0.9–1.34 g/kg BW, *n* = 174), and 1.5 RDA (≥ 1.35 g/kg BW, *n* = 62). After adjusting for age, sex, drinking habit, smoking habit, living condition, and MEQ, PSQI, and IPAQ scores, both the TotalFFM% and AppFFM% of the 0 RDA group were significantly lower than those of the 1 RDA and 1.5 RDA groups (TotalFFM%: 70.1 ± 1.0, 75.1 ± 0.4, and 76.7 ± 0.7%, *p* < 0.001; AppFFM%: 32.1 ± 0.5, 34.1 ± 0.2, and 34.3 ± 0.3%, *p* < 0.001, respectively). On the other hand, there was no significant difference in TotalFFM% and AppFFM% between the 1 RDA and 1.5 RDA groups.

### 3.5. Comparison of Adjusted Protein Intake between AP and NP Groups

We further separated 236 subjects with total protein intake more than the RDA into the AP group (*n* = 83; protein intake over 0.24 g/BW at all three meals) and NP group (*n* = 153; protein intake below 0.24 g/BW in at least one meal out of three meals). Figure 3 illustrates protein intake in total and at each meal in the AP and NP groups after adjusting for age, sex, drinking habit, smoking habit, living condition, PSQI, MEQ, IPAQ scores, and total energy intake. The AP group had significantly higher protein intake in total and at breakfast and lunch than the NP group did (breakfast: 9.7 ± 0.4 in the NP group vs. 17.5 ± 0.5 g in the AP group, *p* < 0.001; lunch: 23.0 ± 0.5 vs. 24.7 ± 0.6 g, *p* = 0.024; dinner: 31.1 ± 0.6 vs. 32.8 ± 0.8 g, *p* = 0.086; snack: 4.4 ± 0.4 vs. 5.1 ± 0.5 kcal, *p* = 0. 274; total: 69.1 ± 1.0 vs. 78.3 ± 1.4 kcal, *p* < 0.001).

### 3.6. Comparison of FFM between AP and NP Groups with Total Protein Intake More Than the RDA

After adjusting for total energy intake with residual methods [7], we selected the subjects with total protein intake more than the RDA and divided them into the AP (*n* = 83) and NP (*n* = 153) groups With adjustment for age, sex, drinking habit, smoking habit, living condition, MEQ, PSQI, and IPAQ scores, the AP group had significantly higher TotalFFM% than the NP group (75.2 ± 0.4 vs. 77.0 ± 0.5, *p* = 0.008), while AppFFM% was possibly higher in the AP group than in the NP group (34.1 ± 0.2 vs. 34.7 ± 0.3, *p* = 0.058) in Figure 4.

## 4. Discussion

In this cross-sectional study, we confirmed that total protein intake more than the RDA (as shown in Figure 2) is essential for the regulation of muscle mass in the healthy young population, but there was no significant difference between the 1 RDA and 1.5 RDA groups. Among individuals consuming total protein intake more than the RDA, we demonstrated that TotalFFM% and AppFFM% in the AP group was greater than those in the NP group. Under free-living conditions, our results suggest that total protein intake is important for the maintenance of muscle mass in individuals with total protein intake below the RDA. On the other hand, among individuals with total protein intake greater than the RDA, achieving 0.24 g/kg BW of protein intake at all three meals could be more effective than total protein intake alone for maintenance of muscle mass.

### 4.1. Influence of Confounding Factors on Outcomes

A previous study conducted among college students confirmed that higher physical activity with the IPAQ score was significantly associated with greater FFM [22]. We also confirmed a positive correlation between physical activity (IPAQ score) and TotalFFM% and AppFFM%, after adjusting for age and sex in our population (data not shown). In addition, smoking habit [16,17,28,29], drinking habit [18,19,30,31], poor sleep quality (higher PSQI scores [20,32,33]) and evening activity preference (fewer MEQ scores [21,34]) were reported to induce unhealthy dietary behaviors. Consequently, those variables may influence protein intake and muscle mass in the present study; therefore, those were included in our analyses as confounding factors.

### 4.2. Importance of Protein Intake More Than the RDA for Regulation of Muscle Mass

To date, total daily protein intake has been mainly focused on for the regulation of muscle mass. A previous cross-sectional study of a population in the mid-life stage in the United States (aged 20–49) has reported that individuals with protein intake greater than the RDA (0.8–1.4 g/kg BW/day) had significantly greater TotalFFM% as compared to those with protein intake below the RDA (<0.8 g/kg BW/day) [11]. However, in that study, TotalFFM% in the highest protein intake group (>1.4 g/kg BW/day) was significantly higher than in the other two groups (<0.8 g and 0.8–1.4 g/kg BW/day groups), while this association was not observed in the present study. This could be due to the differences in the mean ages of the populations in the previous and present studies, as previous reports indicate that aging results in increased protein requirement [14,35]. Although the cut-off points of total protein intake in the previous study (<0.8 g, 0.8–1.4 g, and >1.4 g/kg BW/day) are different from those in the present study (<0.9 g, 0.9–1.34 g, and ≥1.35 g/kg BW/day), we also observed no significant difference in TotalFFM% between the 0.8–1.4 g and >1.4 g/kg groups in our population (data not shown). On the other hand, a previous RCT has demonstrated that there were no significant differences in any anabolic indices (protein expressions or MPS) during the postabsorptive period between among 1, 2, and 3 RDA groups in young subjects (aged 21 ± 1 year) for a 10-day weight maintenance period, a condition in which energy intake equals energy requirement [36]. Considering those findings, protein intake more than the minimum RDA may be necessary for maintaining muscle mass in a young population under free-living conditions.

### 4.3. Effect of Achieving Protein Intake 0.24 g/kg BW at All Three Meals on Muscle Mass

Higher dietary protein intake among three meals was reported to be skewed towards dinner while it was the lowest at breakfast among populations of Japan [37] and the USA [38]. In the present study as well, dietary protein intake was lowest at breakfast and highest at dinner among young subjects. A previous crossover study assessed changes in MPS in response to isoenergetic and isonitrogenous diets with protein at breakfast, lunch, and dinner distributed evenly (EVEN; 0.41, 0.39, and 0.43 g/kg BW of protein intake, respectively) or skewed (SKEW; 0.14, 0.21, and 0.83 g/kg BW of protein intake, respectively) for 7 days [39]. As a result, 24-h MPS at both days 1 and 7 in the EVEN group was significantly higher than that in the SKEW group. The total protein intake in the said study was designed to exceed the RDA (0.8 g/kg BW/day). Interestingly, Moore et al. recently gathered six laboratory-based studies on healthy young and older subjects measuring MPS in response to varying amounts (0–40 g) of high-quality protein as a single bolus using stable isotope methodology [14]. Further statistical analysis indicated 0.24 g/kg BW as the amount of protein needed to maximally increase the MPS with a single protein intake. Considering 0.24 g/kg BW of protein intake for maximization of MPS from a single meal consumption [14], it may support our results that achieving 0.24 g/kg BW of protein intake at all three meals is more effective in maintaining muscle mass than achieving total protein intake more than the RDA.

On the other hand, another study with a longer period (8 weeks) reported different results in older subjects [40]. The randomized control study examining the net protein balance set two groups: SKEW, consuming the majority of protein at dinner (0.16, 0.22, 0.70 g/kg BW of protein intake at breakfast, lunch, and dinner respectively); and EVEN, consuming protein evenly throughout a day (0.36, 0.36, and 0.36 g/kg BW of protein intake, respectively) [40]. In the results after the 8-week intervention, they found no difference in the protein net balance and FFM between the two groups. Notably, the subjects in this previous study were older adults [40], and protein intake for maximization of MPS in older adults (0.40 g/kg BW) was reported to be different from that in young adults (0.24 g/kg BW) [14]. On the contrary, another cross-sectional study investigated the association between protein distribution over three meals and frailty in older subjects with protein intake greater than the RDA, and found that uneven protein distribution was highest in frail subjects compared with pre-frail and non-frail subjects [41]. In older individuals, the effect of protein intake (at three meals) on the anabolic response is still controversial; however, only a few studies have examined the effects, and future interventions are therefore required.

Additionally, for recreationally exercising individuals and athletes, the amount of protein intake at each meal can be greater than that of the general population. For example, a recent systematic review has shown that 1.6 g/kg BW/day of total protein intake was necessary for the resistance training-induced gains in FFM among healthy adults with a resistance training program [42]. Assuming the evenly distributed protein intake, 1.6 g/kg BW of the daily protein intake can be divided into ~0.53 g/kg BW at each meal, and thus the protein intake at each meal for active individuals may be higher than 0.24 g/kg BW. However, there is no study examining the association between protein intake at each meal and muscle mass in those active population; further studies are warranted.

### 4.4. Importance of Protein Intake at Breakfast for the Regulation of Muscle Mass

Breakfast with a high amount of protein may be the key to the regulation of muscle mass under free-living conditions. Most of our subjects achieved 0.24 g/kg BW of protein intake at lunch and dinner but not at breakfast. Our multivariate regression analyses also predicted that achieving 0.24 g/kg BW of protein intake at breakfast could contribute to earning more total daily protein intake (Figure 1). Until now, total protein intake has been well discussed as the main contributor to muscle mass in healthy adults [8,9,11,12,43]. Moreover, older people [10,13,44,45], athletes [46,47,48], and people who undergo resistance training [42] were reported to need more protein than the RDA. Thus, the importance of protein intake at breakfast should be emphasized to achieve the ideal total protein intake. Additionally, our previous study demonstrated a significant association between dietary protein intake and resistance exercise-induced muscle hypertrophy among older adults [49]. Furthermore, there was a significant correlation between protein intake at breakfast and training-induced increase in FFM among those individuals not achieving 0.61 g/kg FFM of daily protein intake. This suggests that protein-rich breakfast is also important for muscle hypertrophy during resistance training, but future studies are warranted among young subjects.

### 4.5. Study Limitations

There are some limitations to our study. First, in this cross-sectional study, we could not fully elucidate the causality between protein intake at three meals and FFM. A further interventional study should be conducted to examine the causality to emphasize the importance of protein intake at three meals. Second, we focused on only energy and macronutrient intakes at three meals (breakfast, lunch, and dinner), except for snacks. This is because the definition of snack is diverse and approximately 20% of our population did not have a snack. In addition, protein intake at snack times was reported to contribute to 1.4% and 2.3% of the total protein intake [50]. Even though the percentage of protein intake at snack time in our population was relatively high as compared with the values in the previous study [50], it was still a minor contribution to total daily protein intake (6.1% in men and 6.7% in women). Third, our results are based on the value of 0.24 g/kg BW derived from a previous study [14]. However, this value may not be applicable to the Japanese population with different dietary habits. Additionally, the cut off value of 0.24 g/kg BW was calculated based on the MPS rates using high-quality protein, such as egg or whey protein, and not mixed meals. Thus, considering the absorption rate into the body between the high-quality protein and regular mixed meals, the value to maximize MPS in mixed meals could be higher. Finally, we used 3-day dietary records to assess dietary intake, although this method was shown to cause under-reporting [51]. However, the dietary records were also reported as a more precise tool than food-frequency questionnaires [52]. To ensure the accuracy of the dietary records, our subjects attended an explanatory meeting about the methodology of noting dietary records before the commencement of the study.

## 5. Conclusions

In conclusion, we elucidated that consuming a total protein intake greater than the RDA and achieving 0.24 g/kg BW of protein intake over three meals is critical for maintaining muscle mass in healthy young subjects under free-living conditions, regardless of other tenable factors, such as sex, physical activity, and total energy intake. In other words, if total protein intake reaches the RDA but protein intake over three meals (breakfast, lunch, and dinner) is not enough, this can result in a negative effect on muscle mass. Therefore, protein intake at each meal, especially breakfast, should be focused on for the regulation of muscle mass.

## Figures and Tables

**Table 1:** Subject characteristics.

	All (*n* = 266)	Men (*n* = 149)	Women (*n* = 117)	*p*-Values
Age (year)	21.4 ± 2.4	21.4 ± 2.3	21.4 ± 2.6	0.381
Drinking habit	79 (29.7)	53 (35.6)	26 (22.2)	0.022
Smoking habit	8 (3.0)	7 (4.7)	1 (0.9)	0.082
Living condition (alone)	163 (61.3)	98 (65.8)	65 (55.6)	0.100
BW (kg)	59.7 ± 10.2	65.8 ± 8.7	52.0 ± 5.9	<0.001
BMI (kg/m^2^)	21.6 ± 2.5	22.4 ± 2.6	20.5 ± 1.8	<0.001
TotalFFM (kg)	45.0 ± 9.9	52.5 ± 5.8	35.4 ± 3.7	<0.001
AppFFM (kg)	20.5 ± 5.2	24.4 ± 3.2	15.5 ± 1.9	<0.001
TotalFFM% (%BW)	74.9 ± 7.8	79.7 ± 6.2	68.9 ± 4.8	<0.001
AppFFM% (%BW)	33.9 ± 4.3	36.9 ± 3.0	30.1 ± 2.3	<0.001
Body fat percentage (%)	21.6 ± 8.2	16.6 ± 6.7	28.0 ± 5.1	<0.001
Sleep condition				
Waking time (h:min)	7:39 ± 1:27	7:54 ± 1:28	7:20 ± 1:24	0.010
Bedtime (h:min)	0:39 ± 1:04	0:45 ± 1:06	0:32 ±1:01	0.143
Sleep latency (min)	25.5 ± 20.9	25.8 ± 20.4	25.1 ± 21.7	0.509
Sleep duration (hour)	6.3 ± 1.3	6.5 ± 1.3	6.1 ± 1.3	0.055
Sleep quality (%)	75.6 ± 17.5	75.2 ± 17.8	76.3 ± 17.2	0.514
PSQI (score)	7.3 ± 2.8	7.0 ± 2.7	7.5 ± 2.9	0.153
MEQ (score)	53.5 ± 7.6	52.7 ± 7.5	54.5 ± 7.6	0.056
IPAQ (MET-min/week)	2721 ± 2438	3298 ± 2571	1987 ± 2044	<0.001
Meal time				
Breakfast time (h:min)	8:45 ± 1:19	8:51 ± 1:23	8:38 ± 1:15	0.478
Lunch time (h:min)	12:51 ± 1:00	12:52 ± 1:07	12:50 ± 0:50	0.968
Dinner time (h:min)	20:21 ± 1:31	20:35 ± 1:35	20:03 ± 1:23	0.004

Values are expressed as means ± SD, or number (%). Abbreviations: TotalFFM, total fat-free mass; AppFFM, appendicular fat-free mass; BW, body weight; PSQI, Pittsburgh Sleep Quality Index; MEQ, Morningness–Eveningness Questionnaire; IPAQ, International Physical Activity Questionnaire. Mann–Whitney *U* test used for continuous variables; chi-squared test used for categorical variables; *p* < 0.05 indicates statistical significance.

**Table 2:** Total dietary intake and dietary intake at each meal.

	All (*n* = 266)	Men (*n* = 149)	Women (*n* = 117)	*p*-Values
Total dietary intake				
Energy (kcal/day)	1933 ± 528	2177 ± 493	1623 ± 393	<0.001
Protein (g/day)	70.2 ± 22.5	80.0 ± 21.6	57.8 ± 16.8	<0.001
Fat (g/day)	66.0 ± 20.4	72.9 ± 21.2	57.2 ± 15.6	<0.001
Carbohydrate (g/day)	255.5 ± 76.7	288.4 ± 75.0	213.8 ± 55.8	<0.001
Protein (g/kg/day)	1.2 ± 0.3	1.2 ± 0.3	1.1 ± 0.4	0.043
Fat (g/kg/day)	1.1 ± 0.3	1.1 ± 0.3	1.1 ± 0.3	0.871
Carbohydrate (g/kg/day)	4.3 ± 1.2	4.4 ± 1.3	4.2 ± 1.2	0.121
Breakfast				
Energy (kcal/meal)	358.5 ± 218.5	380.1 ± 245.8	331.1 ± 175.0	0.118
Protein (g/meal)	12.1 ± 8.6	13.0 ± 9.6	11.0 ± 7.2	0.129
Fat (g/meal)	11.8 ± 8.6	12.3 ± 9.6	11.1 ± 7.1	0.585
Carbohydrate (g/meal)	50.7 ± 31.1	53.6 ± 34.8	46.9 ± 25.3	0.109
Lunch				
Energy (kcal/meal)	647.7 ± 225.8	727.5 ± 211.1	546.0 ± 202.4	<0.001
Protein (g/meal)	23.0 ± 8.9	25.6 ± 8.8	19.8 ± 8.0	<0.001
Fat (g/meal)	21.1 ± 9.3	23.1 ± 9.3	18.6 ± 8.6	<0.001
Carbohydrate (g/meal)	88.0 ± 33.3	100.1 ± 32.4	72.7 ± 27.6	<0.001
Dinner				
Energy (kcal/meal)	750.2 ± 305.8	898.6 ± 283.5	561.1 ± 216.3	<0.001
Protein (g/meal)	30.6 ± 13.7	36.5 ± 13.1	23.1 ± 10.4	<0.001
Fat (g/meal)	27.0 ± 13.1	31.9 ± 13.1	20.6 ± 10.0	<0.001
Carbohydrate (g/meal)	90.7 ± 40.6	109.1 ± 39.4	67.2 ± 28.1	<0.001
Snack				
Energy (kcal/meal)	177.0 ± 170.1	171.2 ± 180.6	184.5 ± 156.1	0.130
Protein (g/meal)	4.5 ± 5.7	4.9 ± 6.7	3.9 ± 4.0	0.450
Fat (g/meal)	6.1 ± 7.1	5.5 ± 7.5	6.9 ± 6.5	0.002
Carbohydrate (g/meal)	26.2 ± 25.5	25.5 ± 27.2	27.0 ± 23.3	0.144

Values are expressed as means ± SD. Mann–Whitney *U* test used for continuous variables; *p* < 0.05 indicates statistical significance.

## Supplementary Materials

The following are available online at https://www.mdpi.com/2072-6643/11/3/612/s1, Table S1: Subject characteristics between the NP and AP groups in men, Table S2: Subject characteristics between NP and AP groups in women, Table S3: Total dietary intake and dietary intake at each meal between NP and AP groups in men, Table S4: Total dietary intake and dietary intake at each meal between NP and AP groups in women.
