# Supplementary material for: Association of Protein Intake in Three Meals with Muscle Mass in Healthy Young Subjects: A Cross-Sectional Study
Source: Nutrients. 2019 Mar 13;11(3):612. doi: 10.3390/nu11030612 (PMC6471574; doi:10.3390/nu11030612)
Supplement: Supplementary file 1 [file nutrients-11-00612-s001.zip › nutrients-417117-SI.pdf]

Table S1. Subject characteristics between NP and AP groups in men.

	NP (n = 104)				AP (n = 45)				P values
Age (yr)	21.7	±	2.4		20.9	±	2.2		0.070
Drinking habit	38	(	36.5	)	15	(	33.3	)	0.852
Smoking habit	6	(	5.8	)	1	(	2.2	)	0.675
Living condition (alone)	75	(	72.1	)	23	(	51.1	)	0.015
Weight (kg)	66.4	±	9.8		66.1	±	6.7		0.549
BMI (kg/m^2)	22.4	±	2.8		22.4	±	2.1		0.395
TotalFFM (kg)	52.1	±	6.1		53.5	±	5.0		0.037
AppFFM (kg)	24.2	±	3.4		24.9	±	2.7		0.061
%TotalFFM (% weight)	79.0	±	6.5		81.2	±	5.3		0.048
%AppFFM (% weight)	36.5	±	3.1		37.7	±	2.5		0.018
Body fat percentage (%)	17.3	±	6.9		15.0	±	5.7		0.040
Sleep condition									
Waking time (h:min)	7:57	±	1:20		7:48	±	1:43		0.320
Bedtime (h:min)	0:45	±	1:06		0:44	±	1:08		0.762
Sleep latency (min)	26.7	±	21.9		23.6	±	16.6		0.667
Sleep duration (hour)	6.5	±	1.2		6.5	±	1.4		0.850
Sleep quality (%)	74.5	±	17.3		76.7	±	19.1		0.455
PSQI (score)	7.2	±	2.6		6.7	±	2.8		0.341
MEQ (score)	52.2	±	7.5		53.9	±	7.4		0.105
IPAQ (MET-min/week)	3059	±	2327		3850	±	3020		0.222
Meal time									
Breakfast time (h:min)	8:59	±	1:11		8:34	±	1:41		0.016
Lunch time (h:min)	12:50	±	0:49		12:57	±	1:35		0.614
Dinner time (h:min)	20:38	±	1:36		20:28	±	1:33		0.349

Values are expressed as means ± SD, or number (%). Abbreviations: TotalFFM, total fat-free mass; AppFFM, appendicular fat-free mass; BW, body weight; PSQI, Pittsburgh sleep quality index; MEQ, morningness-eveningness questionnaire; IPAQ, international physical activity questionnaire.
Mann–Whitney U test used for continuous variables; Chi-square test used for categorical variables; $P < 0.05$ indicates statistical significance.

Table S2. Subject characteristics between NP and AP groups in women.

	NP (n = 86)				AP (n = 31)				P values
Age (yr)	21.4	±	2.5		21.4	±	2.9		0.563
Drinking habit	19	(	22.1	)	7	(	22.6	)	1.000
Smoking habit	1	(	1.2	)	0	(	0.0	)	1.000
Living condition (alone)	53	(	61.6	)	12	(	38.7	)	0.035
Weight (kg)	52.3	±	5.8		49.6	±	5.7		0.013
BMI (kg/m^2)	20.6	±	1.7		20.2	±	1.9		0.141
TotalFFM (kg)	35.8	±	3.8		34.3	±	3.4		0.032

AppFFM (kg)	15.7	±	2.0	14.9	±	1.6	0.023
%TotalFFM (% weight)	68.6	±	4.8	69.4	±	4.5	0.243
%AppFFM (% weight)	30.1	±	2.4	30.2	±	2.0	0.613
Body fat percentage (%)	28.2	±	5.2	27.4	±	4.8	0.277
Sleep condition							
Waking time (h:min)	7:28	±	1:18	6:57	±	1:35	0.076
Bedtime (h:min)	0:37	±	0:54	0:17	±	1:17	0.095
Sleep latency (h:min)	27.7	±	22.8	17.9	±	16.5	0.018
Sleep duration (h:min)	6.1	±	1.4	6.1	±	1.2	0.948
Sleep quality (%)	74.8	±	16.7	80.3	±	18.1	0.049
PSQI (score)	7.8	±	2.9	6.8	±	2.7	0.099
MEQ (score)	54.3	±	7.1	54.9	±	9.1	0.865
IPAQ (MET-min/week)	2062	±	2256	1779	±	1291	0.632
Meal time							
Breakfast time (h:min)	8:43	±	1:14	8:26	±	1:17	0.156
Lunch time (h:min)	12:47	±	0:43	12:56	±	1:04	0.709
Dinner time (h:min)	20:05	±	1:31	19:55	±	0:57	0.870

Values are expressed as means ± SD, or number (%). Abbreviations: TotalFFM, total fat-free mass; AppFFM, appendicular fat-free mass; BW, body weight; PSQI, Pittsburgh sleep quality index; MEQ, morningness-eveningness questionnaire; IPAQ, international physical activity questionnaire.
Mann–Whitney U test used for continuous variables; Chi-square test used for categorical variables; $P < 0.05$ indicates statistical significance.

Table 3. Total dietary intake and dietary intake at each meal between NP and AP groups in men.

	NP (n = 104)			AP (n = 45)			P values
Total dietary intake							
Energy (kcal/day)	2051	±	453	2468	±	461	< 0.001
Protein (g/day)	72.7	±	17.8	97.0	±	20.0	< 0.001
Fat (g/day)	68.4	±	19.5	83.3	±	21.5	< 0.001
Carbohydrate (g/day)	274.7	±	70.2	319.8	±	77.1	0.001
Protein (g/kg/day)	1.1	±	0.3	1.5	±	0.3	< 0.001
Fat (g/kg/day)	1.0	±	0.3	1.3	±	0.3	< 0.001
Carbohydrate (g/kg/day)	4.2	±	1.3	4.9	±	1.2	0.002
Breakfast							
Energy (kcal/meal)	283.4	±	206.7	603.3	±	173.5	< 0.001
Protein (g/meal)	8.3	±	6.5	23.7	±	6.5	< 0.001
Fat (g/meal)	8.6	±	7.5	20.8	±	8.5	< 0.001
Carbohydrate (g/meal)	42.6	±	31.7	79.2	±	27.9	< 0.001
Lunch							
Energy (kcal/meal)	709.2	±	218.2	769.6	±	189.4	0.271

Protein (g/meal)	24.2	±	8.4	28.8	±	8.8	0.006
Fat (g/meal)	22.9	±	9.3	23.7	±	9.5	0.830
Carbohydrate (g/meal)	97.6	±	32.9	105.9	±	30.9	0.165
Dinner							
Energy (kcal/meal)	887.7	±	298.2	923.6	±	247.4	0.503
Protein (g/meal)	35.4	±	13.2	39.1	±	12.7	0.193
Fat (g/meal)	31.2	±	13.0	33.7	±	13.5	0.392
Carbohydrate (g/meal)	109.2	±	41.3	108.8	±	35.1	0.885
Snack							
Energy (kcal/meal)	171.0	±	173.6	171.6	±	197.7	0.761
Protein (g/meal)	4.7	±	6.6	5.4	±	7.0	0.797
Fat (g/meal)	5.7	±	7.8	5.1	±	6.9	0.705
Carbohydrate (g/meal)	25.4	±	24.6	26.0	±	32.7	0.455

Values are expressed as means ± SD. Mann–Whitney U test used for continuous variables; P < 0.05 indicates statistical significance.

Table S4. Total dietary intake and dietary intake at each meal. between NP and AP groups in women.

	NP (n = 86)			AP (n = 31)			P values
Total dietary intake							
Energy (kcal/day)	1523	±	375	1899	±	303	< 0.001
Protein (g/day)	53.0	±	15.1	71.0	±	14.2	< 0.001
Fat (g/day)	54.1	±	15.4	65.6	±	12.9	< 0.001
Carbohydrate (g/day)	200.7	±	54.2	250.1	±	43.1	< 0.001
Protein (g/kg/day)	1.0	±	0.3	1.4	±	0.3	< 0.001
Fat (g/kg/day)	1.0	±	0.3	1.3	±	0.3	< 0.001
Carbohydrate (g/kg/day)	3.9	±	1.0	5.1	±	1.0	< 0.001
Breakfast							
Energy (kcal/meal)	272.4	±	150.2	494.0	±	131.1	< 0.001
Protein (g/meal)	8.3	±	5.9	18.4	±	4.9	< 0.001
Fat (g/meal)	8.8	±	5.9	17.4	±	6.4	< 0.001
Carbohydrate (g/meal)	40.0	±	21.8	65.8	±	24.8	< 0.001
Lunch							
Energy (kcal/meal)	531.1	±	217.4	587.4	±	148.6	0.451
Protein (g/meal)	19.1	±	8.6	21.6	±	5.8	0.165
Fat (g/meal)	18.5	±	9.5	18.7	±	5.1	0.961
Carbohydrate (g/meal)	69.5	±	28.3	81.5	±	23.8	0.068
Dinner							
Energy (kcal/meal)	529.9	±	214.2	647.8	±	200.7	0.022
Protein (g/meal)	21.7	±	10.5	27.3	±	8.8	0.014

Fat (g/meal)	19.6	±	9.7	23.6	±	10.4	0.052
Carbohydrate (g/meal)	63.6	±	28.8	77.1	±	23.7	0.020
Snack							
Energy (kcal/meal)	190.0	±	168.0	169.4	±	118.4	0.938
Protein (g/meal)	3.9	±	4.2	3.8	±	3.3	0.743
Fat (g/meal)	7.2	±	7.0	5.9	±	5.0	0.532
Carbohydrate (g/meal)	27.6	±	24.8	25.6	±	18.4	0.995

Values are expressed as means ± SD. Mann–Whitney U test used for continuous variables; $P < 0.05$ indicates statistical significance.
